# Association of supragingival plaque management with subgingival microbiota is moderated by adjunctive antibiotics in stage III-IV periodontitis patients during periodontal therapy

**DOI:** 10.1080/20002297.2025.2517043

**Published:** 2025-06-14

**Authors:** Kyana Charlotte Laura Saberi Kakhki, Inga Harks, Johannes Matern, Karola Prior, Peter Eickholz, Katrin Lorenz, Ti-Sun Kim, Thomas Kocher, Jörg Meyle, Doğan Kaner, Yvonne Jockel-Schneider, Dag Harmsen, Benjamin Ehmke, Sven Kleine Bardenhorst, Daniel Hagenfeld

**Affiliations:** a Department of Periodontology and Operative Dentistry, MünsterUniversity Hospital, Münster, Germany; b Department of Periodontology, Center for Dentistry and Oral Medicine, Goethe-University Frankfurt, Frankfurt, Germany; c Department of Periodontology, TU Dresden, Dresden, Germany; d Section of Periodontology, Department of Conservative Dentistry, Clinic for Oral, Dental and Maxillofacial Diseases, Heidelberg University Hospital, Heidelberg, Germany; e Department of Restorative Dentistry, Periodontology, Endodontology, and Preventive and Paediatric Dentistry, University Medicine Greifswald, Greifswald, Germany; f Department of Periodontology, University of Giessen, Giessen, Germany; g Departments of Periodontology and Synoptic Dentistry, Charite-Universitätsmedizin Berlin, Berlin, Germany; h Department of Periodontology, Dental School, Faculty of Health, University of Witten/Herdecke, Witten, Germany; i Department of Periodontology, University Hospital Würzburg, Würzburg, Germany; j Institute of Epidemiology and Social Medicine, University of Münster, Münster, Germany

**Keywords:** Dental plaque, microbiota, dysbiosis, periodontal debridement, adjunctive antibiotics

## Abstract

**Background:**

This study examines the relationship between supragingival plaque control and subgingival microbiota during periodontal therapy, focusing on microbial clusters associated with plaque levels.

**Methods:**

Data were drawn from a 26-month multicenter, double-blinded, randomized, placebo-controlled trial. Supragingival plaque was measured using the O’Leary index, and subgingival microbiota were profiled via Illumina 16S rRNA gene sequencing. A novel topic modelling approach using cross-validated Latent Dirichlet Allocation (LDA) identified microbial clusters, and negative binomial mixed models evaluated their association with plaque levels.

**Results:**

Supragingival plaque was positively associated with bleeding on probing (BOP) and microbial diversity, but not with dysbiosis. A specific subgingival microbial cluster dominated by *Selenomonas* and *Leptotrichia* was linked to elevated plaque levels and increased in abundance following both antibiotic and placebo treatments. The odds ratio for plaque associated with this cluster was 1.20 (95% CI: 1.07–1.35). Stratified analyses showed this association was reduced in the antibiotic group but remained in the placebo group.

**Conclusion:**

Ineffective supragingival plaque control correlates with increased BOP and microbial diversity, though not necessarily with dysbiosis. Adjunctive antibiotics may promote a more cariogenic subgingival microbiota by disrupting the association between plaque accumulation and the abundance of acidogenic taxa such as *Selenomonas* and *Leptotrichia*.

## Introduction

The routine removal of dental biofilm is essential in the prevention and treatment of periodontitis. The ‘Treatment of stage I – III periodontitis – The EFP S3 level clinical practice guideline’ emphasizes that effective periodontal therapy begins with providing patients with preventive and health promotion tools, as well as supragingival dental biofilm control [1]. As the first stage of periodontal therapy, this ‘pre-treatment’ includes motivating patients and encouraging behavioural changes to improve self-performed oral hygiene to reduce supragingival plaque levels [2,3]. Crucially, pre-treatment also involves the professional mechanical removal of supragingival plaque and calculus, together with the elimination of local retentive factors, identified as professional mechanical plaque removal (PMPR). Although this initial pre-treatment alone is insufficient to treat periodontitis, it should set the stage for optimal treatment response and secondary prevention of periodontitis [1,4]. In clinical scenarios, such as when deep probing depths are present, the initial and secondary phases of therapy may be administered concurrently, also to prevent the development of periodontal abscesses [1]. The accumulation of supragingival plaque is thought to lead to a bacterial reservoir that may contribute to the subsequent transition from a periodontally healthy to a periodontally diseased individual [5].

While it is well-established that supragingival plaque can influence subgingival microbial communities, the potential for subgingival bacteria to migrate into the supragingival biofilm is less understood [6]. Studies have indicated that pathogenic bacteria residing in subgingival niches may recolonize supragingival areas, promoting biofilm formation and potentially undermining the effectiveness of supragingival plaque control measures [7,8]. This highlights a possible bidirectional relationship between these microbial ecosystems, where subgingival pathogens may also affect supragingival biofilm composition.

Studies have shown that rigorous supragingival plaque control performed during and after scaling and root planing (SRP) significantly improves periodontal treatment outcomes [9]. Weekly professionally administered supragingival plaque removal has been demonstrated to markedly reduce both supra- and subgingival bacterial counts in the subgingival biofilm [10]. Adjunctive treatments with professional mechanical plaque control or chlorhexidine rinsing have shown greater clinical and microbiological benefits compared to SRP alone [11]. These findings underscore the pivotal role of supragingival plaque management in reducing pathogenic bacterial species and improving clinical parameters [12–17]. Those studies, however, have rather small sample sizes and a short follow up. We hypothesize that the effective management of supragingival plaque is also associated with distinct alterations in the subgingival microbiota during periodontal therapy.

The aim of this study is to investigate the relationship between supragingival plaque management and the composition of subgingival microbiota in a long-term study with larger sample size on periodontal therapy in stage III – IV periodontitis patients.

## Material and methods

### Study design

This study is an extension of previously published research that investigated microbial outcomes of periodontal therapy [18–20]. Specimens from the ABPARO study were analysed that is a multicenter, randomized, double-blinded, parallel-group, and placebo-controlled trial investigating the effects of adjunctive antibiotics during periodontal treatment (ISRCTN: 64254080, ClinicalTrials.gov: NCT00707369). Periodontitis patients which have a medication affecting the periodontium and patients receiving periodontal treatment with and without antibiotics 6 months prior to this study were excluded. All patients were anonymized for this secondary analysis. This research adheres to the ethical principles outlined in the World Medical Association’s Declaration of Helsinki, revised in October 2013, and received approval from the ethics committee of the University of Muenster, Germany (2016–505-f-S).

The original ABPARO study included 345 patients, who adhered strictly to the treatment protocol (per-protocol stratum). For this secondary analysis, 163 patients were selected from the per-protocol stratum as previously described [20], including 82 who received non-surgical periodontal therapy with systemic adjunctive placebo (PL) and 81 who received systemic adjunctive antibiotics (AB). Plaque samples were available for all patients. Detailed study specifications can be found elsewhere [21–24].

Patients underwent mechanical therapy within six weeks (visit 3/V3) after baseline measurements (visit 2/V2), which included supra- and subgingival debridement using sonic/ultrasonic scalers, hand instruments and air powder devices under local anaesthesia. Subsequently, patients received either study medication (amoxicillin 3H2O 574 mg [Amoxicillin-Ratiopharm 500 mg, Ratiopharm, Germany]; metronidazole 400 mg [Flagyl 400, Sanofi-Aventis, Germany]) or identical looking placebo pills, taken three times daily for seven days. Supportive periodontal treatment followed after two months (visit 4/V4), including full-mouth supragingival debridement and oral hygiene instructions at three-month intervals for 26 months (visit 5–12/V5–V12). Participants received oral hygiene instructions at baseline and at each supportive visit. Periodontal measurements were obtained for each patient at six sites per tooth. The assessments included the percentage of sites with a probing depth of 5 mm or more (%PPD ≥5 mm), the percentage of sites exhibiting bleeding on probing (%BOP), and the percentage of sites showing additional clinical attachment loss of at least 1.3 mm following mechanical therapy (%PSAL). All measurements were conducted by examiners blinded to the study conditions. The relative amount of visible supragingival plaque was investigated using the O’Leary index after staining the tooth with Mira-2-Ton (Hager and Werken, Duisburg Germany) at baseline, and at 2, 8, 14 and 26 months after periodontal therapy. The O’Leary index was calculated as relative proportions, assessing plaque presence on mesial, buccal, distal and lingual surfaces of each tooth. Sites with PPD ≥4 mm received additional subgingival re-debridement during supportive periodontal therapy. Subgingival specimens were collected from the deepest pocket per quadrant (probing depth ≥6 mm), using a sterile paper point inserted for 10 seconds at baseline and at 2, 8, 14 and 26 months. All samples collected during one visit were pooled to ensure a homogeneous distribution of diseased sampling sites [18,25].

### Library preparation, sequencing, and bioinformatics

Bacterial genomic DNA was isolated and purified using a QIAamp Mini DNA Isolation Kit as previously described [19]. Sequencing, library preparation and PCR protocols mirrored those used in our previous studies. Briefly, the 16S rRNA gene’s hypervariable region V4 was targeted using two PCRs performed with KAPA HiFi HotStart Ready Mix. The first PCR used universal bacterial primers targeting V4 regions, followed by a second PCR with sample-specific barcodes. Up to 96 libraries were normalized and pooled for sequencing on an Illumina MiSeq platform, generating 250 base pair paired-end reads. Sequencing runs included negative controls and a balanced mock sample. Raw data are available from the European Nucleotide Archive under accession number PRJEB51017.

Bioinformatic analysis was performed using Illumina’s MiSeq Control Software v2.6.2.1, Real-Time Analysis v1.18.54, MiSeq Reporter v2.6.3, and Cutadapt v1.8.1 [26]. Sequences were processed using R v4.0.4 [27] and RStudio v1.1.463 [28], with DADA2 v1.20.0 [29] for quality filtering and identification of amplicon sequence variants (ASVs). Low-quality reads and chimeras were removed, and taxonomy was assigned using the native naive Bayesian classifier of DADA2 [29] with the eHOMD v. 15.23 [30] and SILVA reference database v1.15.1 [31] as previously described. The subgingival microbiome composition was described by alpha-, beta-diversity indices [32] and subgingival dysbiosis measured by the genus-level subgingival dysbiosis index (SMDI) [33], and as dysbiosis topic as described in our previous publication [34]. Alpha diversity is a measure of the diversity of an ecological niche and can be measured as observed RSVs for richness. The Beta diversity measure we applied was Bray-Curtis sample dissimilarity.

### Statistical analysis

**Figure 1.**
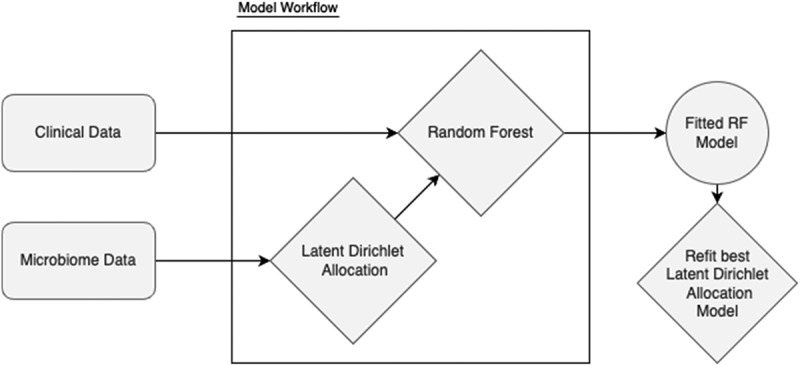
Workflow of machine learning pipeline. The model workflow incorporates hyperparameter optimization and cross-validation across all steps included in the workflow. Rounded rectangles, diamonds and circles represent raw data inputs, model fitting and fitted models, respectively.

The current analysis introduces new data on supragingival plaque levels, measured using the O`Leary index [35], and incorporates a pipeline utilizing topic modelling [36] that we introduced in our previous publication [34] to evaluate its association with subgingival bacterial composition. The study population was described descriptively to find potential differences of supragingival plaque levels across visits, different study centers, treatment groups, age, sex and smoking status at baseline. To find potential associations of the oral microbiome with supragingival plaque levels (as measured by the O’Leary index) we created explorative univariate linear models looking at alpha- and beta-diversity, genus-level SMDI and the main dysbiosis topic, corrected for multiple comparisons (FDR). Furthermore, we implemented a pipeline to detect microbial sub-communities that are robustly associated with increased sites with supragingival plaque levels. In particular, we implemented a novel cross-validated machine learning pipeline combining Latent Dirichlet Allocation (LDA) and Random Forest regression analysis [34] (Figure 1). In comparison to other clustering approaches, LDA does not assume fixed cluster membership, but models each sample as a mixture of different sub-communities, thus detected clusters can be present in varying proportions across samples, better reflecting the complex ecological nature of microbiome data. Microbial abundance data at the genus level was obtained from baseline samples and preprocessed using a custom dimensionality reduction step based on LDA. Note, that the LDA step is not precalculated but part of the model building process, thus, cross-validated. The pipeline was implemented in R using the tidymodels framework, with a workflow that incorporated both feature engineering and model training. The Random Forest model was tuned across multiple hyperparameter combinations, including the number of trees (500–2000), number of variables randomly sampled as candidates at each split (mtry: 5–15), minimum node size (1–15), and the number of LDA topics (5–30). Model performance was evaluated using 5-fold cross-validation to ensure robust estimation of association. Hyperparameter optimization was performed using a grid search approach across 360 different parameter combinations. The Random Forest model was implemented using the ranger engine with impurity-based variable importance calculations. Baseline data (before treatment) was used to ensure microbial composition was not biased by periodontal treatment. From the best performing model, the microbial sub-community (topic) with the highest importance measure was selected as candidate topic and further evaluated on the entire data.

A mixed logistic regression model with a nested random effect to account for the longitudinal nature of the data and sampling at different study centers was used. The model was adjusted for confounders including age, sex, smoking status, antibiotic treatment as well as clinical parameters. We further adjusted for the number of pre-treatments, which varied depending on the study center, as data suggested a strong influence on baseline plaque levels. Additionally, the dysbiotic main topic [34] was considered to differentiate the general effects of dysbiosis from the specific impacts of the identified topic. As antibiotic treatment is expected to strongly influence the microbiome-outcome association, we performed stratified analyses of the final model within the treatment groups to assess the robustness of the detected associations.

## Results

### Inter-center variability in pre-treatment frequency and baseline plaque levels

The study included participants from eight study centers, each with varying frequencies of pre-treatments. Würzburg that conducted no pre-treatments had a median O´Leary value of 33.8%, while Berlin carried out an average of five pre-treatment session, had a median O´Leary of 9.6%. The other study centers performed an average of two pre-treatments and had O´Leary values between 21.0% in Frankfurt and 67.6% in Giessen. Other demographic variables such as smoking status, age, sex, as well as the treatment group at baseline (before administering adjunctive placebo vs. antibiotics) did not show noticeably different plaque values at baseline.

### Associations between supragingival plaque, bleeding on probing and microbial diversity

**Figure 2.**
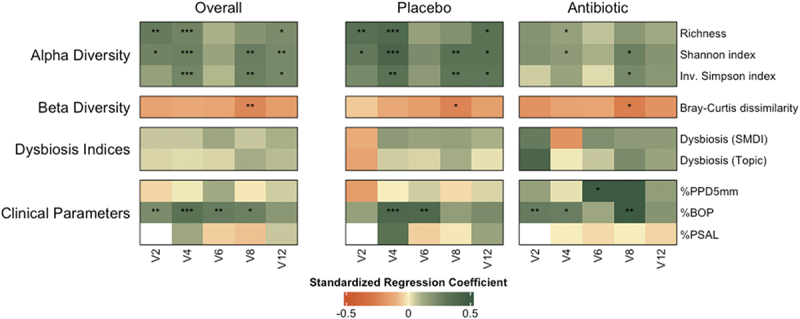
Univariate associations between microbial and clinical variables and supragingival plaque levels across timepoints. Heatmap displays standardized regression coefficients from univariate regression models, stratified by treatment group (overall, placebo, antibiotic). SMDI was calculated on genus level as described by Chen et al. [33] and dysbiosis topic as previously described [34]. Color intensity reflects the magnitude and direction of the standardized regression coefficients, with significance levels denoted as *p < 0.05, **p < 0.01, and ***p < 0.001.

The association between subgingival microbiota indices and clinical indices with supragingival plaque values was assessed using explorative univariate models at each timepoint (Figure 2, Supp. Table 1).

**Table 1.** Descriptive analysis of the supragingival plaque value in relation to study specific characteristics at baseline.

Characteristics		O´Leary value
Study center		Median% (25%/75% quantile)
	Berlin (*N* = 40)	9.6 (5.3/18.8)
	Dresden (*N* = 21)	48.2 (30.0/60.7)
	Frankfurt (*N* = 39)	21.0 (14.2/29.6)
	Giessen (*N* = 36)	67.6 (29.8/83.1)
	Greifswald (*N* = 20)	23.7 (16.5/43.0)
	Heidelberg (*N*=23)	56.3 (35.7/71.2)
	Münster (*N* = 98)	36.8 (20.6/52.3)
	Wuerzburg (*N* = 68)	33.8 (20.4/48.0)
Treatment group		
	placebo (*N* = 82)	30.2 (16.1/52.01)
	antibiotics (*N* =81)	36.0 (20.8/55.8)
Sex		
	female (*N* = 83)	31.3 (14.7/50.0)
	male (*N* = 80)	34.2 (21.8/57.4)
Age		
	<45 (*N*= 36)	25.3 (12.2/45.5)
	≤45<55 (*N*= 65)	33.9 (16.7/50.0)
	≥55 (*N* = 62)	37.5 (20.8/59.7)
Smoking		
	non-smoker (*N* = 94)	32.4 (18.6/57.7)
	smoker (*N* = 96)	34.1 (16.1/49.1)

Supragingival Plaque was measured by O´Leary index [35] .

Regarding overall clinical indices , Bleeding on Probing (BOP) was positively associated with O’Leary values before therapy(E = 0.31, 95% CI: 0.13–0.49, adj. *p* < 0.009). This association remained statistically significant until 14 months after periodontal therapy. At 26 months BOP no longer was statistically significantly associated with BOP. Pocket probing depth (%PPD5mm) and attachment loss (PSAL  ) were not significantly associated with plaque values at any timepoint.

#### Adjunctive antibiotics reduce bleeding on probing and weakens its association with plaque levels

**Figure 3.**
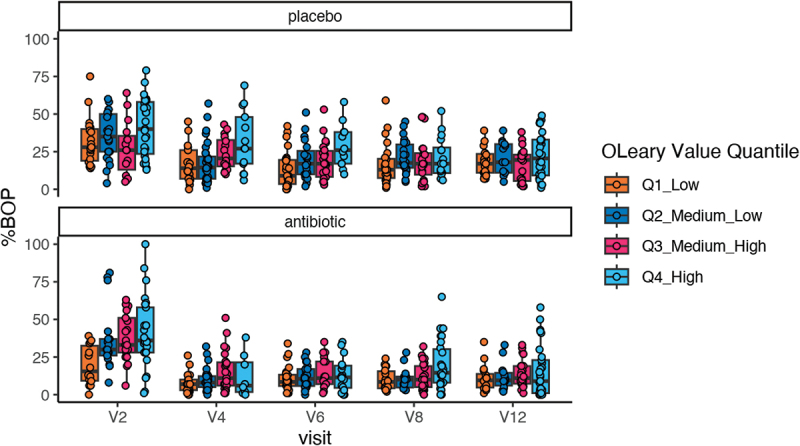
Box plots illustrating the percentage of bleeding on probing (%BOP) measured across study visits V2 (baseline) followed by V4 (at 2 months), V6 (at 8 months), V8 at (at 14 months) and V12 (at 26 months after periodontal therapy). Data points are stratified by O’Leary index quantiles.

Stratified analyses by treatment group revealed weaker effect sizes in the antibiotic group compared to placebo, with no clear pattern in significance levels. To further explore this associations the BOP and Quantiles of the O´Leary indices were plotted together for each study visit (Figure 3). Figure 3 reveals that before therapy, increasing O´Leary Quantiles were associated with increased BOP. For BOP a clear treatment effect of adjunctive antibiotics can be seen as BOP was reduced at V4 and remains mostly under 25% irrespective of increasing O`Leary values, except for a few outliers with higher BOP in the 4^th^ O´Leary Quantile at V8. The treatment effect of adjunctive placebo on BOP seems to occur at a later timepoint (V8-V12) were increasing O´Leary quantiles did not lead to a higher BOP.

#### Microbial diversity and not dysbiosis is associated with plaque levels

Regarding microbiome indices before therapy (V2), a higher proportion of tooth sites with visible supragingival plaque (O’Leary) was strongly associated with elevated alpha diversity in terms of species richness (Estimate (E) = 0.21, adjusted p-value (adj.*p*) = 0.0025) and Shannon index (E = 12.37 adj.*p* = 0.02). Two months after therapy (V4), all alpha diversity were significantly linked to supragingival plaque levels (richness: E = 0.19, adj.*p* < 0.001, Shannon: E = 13.1, adj.*p* < 0.001. Inv.Simpson: E = 0.6, adj.*p* < 0.001). This association was not observed for V6, but was reestablished from V8-V12 for Shannon and Simpson indices (E = 11.73, adj.*p* < 0.01; E = 0.58, adj.*p* < 0.05). At V8 (14 months after therapy), beta diversity (mean Bray-Curtis distances) showed a negative association with plaque values (E = −95.31, adj.*p* < 0.01), but was not significantly associated at any other time point. The stratified analyses revealed a strong influence of adjunctive antibiotics on the association of diversity with plaque levels. Dysbiosis, however, regardless if, measured as SMDI or as dysbiosis-topic did not reveal significant associations at any visit and no noticeable additional effect if adjunctive placebo or antibiotics were given.

### Identification of a plaque-associated microbial topic and its modulation by antibiotic therapy

As higher microbial diversity but not dysbiosis was associated with plaque values we explored other potential microbial clusters within the subgingival microbiome that might be associated with O´Leary values. To look for any other clusters, *i.e*. topics, within the microbiota that are associated with increasing plaque-values, we created a topic model with 19 possible topics as indicated as best model by our topic modelling pipeline (Suppl. Figure S1) including one specific microbial topic, showing the highest association with the O´Leary value. *Selenomonas* and *Leptotrichia* dominate this topic, showing the highest contributions with values close to 40% in relation to other bacteria representing this topic. Following these, *Saccharibacteria* (TM7) [G-1], *Capnocytophaga*, *Corynebacterium*, *Campylobacter*, *Prevotella*, *Lachnoanaerobaculum*, and *Tannerella* are also defining this topic, displaying substantially lower contributions (Suppl. Figure S2).

The temporal dynamics of the genera *Leptotrichia* and *Selenomonas* differed between treatment groups over the study period (Suppl. Figure S3). In the antibiotic group, *Leptotrichia* exhibited a sharp increase in relative abundance two months after therapy (V4), followed by a gradual decline that plateaued through 26 months after therapy (V12). In contrast, the placebo group showed a slower increase, peaking at eight months after therapy, with a subsequent modest decline. Similarly, *Selenomonas* abundance rose rapidly in the antibiotic group, peaking at two months after therapy (V4), then progressively decreased over time. The placebo group displayed a more muted peak and a comparable downward trend, consistently maintaining lower abundance levels than the antibiotic group across all visits.

For each sample, contribution of the *Selenomonas/Leptotrichia* topic was extracted, and the effects of study-center and BOP stratified by placebo and antibiotic group were further investigated using a negative binomial mixed model (Table 2).

**Table 2.** Effects of O’Leary plaque index (in 10% increments) on shares of Selenomonas/Leptotrichia (below/above median).

	Overall		Antibiotics		Placebo	
Characteristic	OR	95% CI	OR	95% CI	OR	95% CI
O´Leary value	1.20	1.07, 1.35	1.10	0.95, 1.29	1.21	1.00, 1.47

The presented odds ratios (OR) along with their 95% credible intervals (CI) were derived from a mixed effects logistic regression model. The model included random effects for individual participants and study centers to account for repeated sampling of individuals across multiple visits and clustering within different study sites. ORs are adjusted for the confounding factors age, sex, smoking status, visit, pre-treatment, dysbiosis, % sites with bleeding on probing, % of sites with PPD >5 mm, and % of site with clinical attachment loss >1.3 mm.

The overall odds ratio (OR) for the O´Leary value was 1.20 meaning that an increase in 10% O´Leary value leads to 20% increased chance for the occurrence of this microbial topic. In the adjunctive antibiotic group the odds ratio was lower at 1.10 and not statistically significant, whereas for the placebo group a similar OR of 1.21 was observed that was statistically significant. This shows a possible moderating effect of the adjunctive antibiotics on the prediction of the *Selenomonas/Leptotrichia* topic by O´Leary values.

To explore different *Selenomonas* and *Leptotrichia* species during periodontal therapy with adjunctive antibiotics and placebo a sensitivity analysis for eight *Selenomonas* species and seven *Leptotrichia* species was conducted (Suppl. Fig S4). Here no noticeable differences of any species were found showing similar abundances over the course of periodontal therapy.

## Discussion

This study aimed to evaluate the association of O`Leary values and the oral microbiota during periodontal therapy, with a specific focus on identifying subgingival microbial topics associated with supragingival plaque levels. Overall higher Plaque values were associated with higher richness and BOP but not with subgingival dysbiosis. Our topic analysis revealed that higher O`Leary values predict the occurrence of a microbial topic dominated by *Selenomonas* and *Leptotrichia*. This association was diminished by adjunctive antibiotics during periodontal therapy.

Our initial exploratory analysis reveals a significant positive association between BOP and alpha diversity (richness, Shannon diversity and inverse Simpson diversity) with supragingival plaque levels. A secondary analysis of a randomized clinical trial by Reiniger et al., 2021 [37] that included 42 subjects over a 90-day period revealed that stronger correlations between plaque index and bleeding on probing were observed with extended oral hygiene intervals. Higher O`Leary values may facilitate the colonization of additional bacteria in the subgingival plaque at the beginning and during periodontal therapy. However, these newly colonized bacteria may not significantly contribute to dysbiosis as neither SMDI nor dysbiotic-topic was associated with higher plaque levels (Figure 2). An alternative explanation is that reducing both supragingival and subgingival plaque levels decreases the periodontal bleeding and pocket depth, thereby reducing total bacterial load without necessarily shifting the microbial community towards a more normobiotic state. This implies that while plaque reduction is beneficial for gingival health and caries prevention, it may not significantly alter the dysbiotic condition of the subgingival microbiome. As shown in our previous publications, however, reducing the subgingival dysbiosis greatly influences long-term periodontal stability [20,34]. Therefore, interventions targeting plaque reduction should be complemented with strategies aimed at modifying the microbial environment to achieve more favourable clinical outcomes.

In the second part of this manuscript we explored potential microbial clusters associated with high plaque levels employing a pipeline using LDA and Random Forest modelling (Figure 1). We found a subgingival topic dominated by *Selenomonas* and *Leptotrichia* not being typically considered periodontal pathobionts. However, their involvement in caries development [38], especially in supragingival plaque, is documented [39]. This observation also aligns with previous reports [40,41] demonstrating an ecological shift toward cariogenic species when periodontal pathogens are reduced. In this context, our data support the findings of Ravald et al [42], who showed that patients treated for periodontitis remained vulnerable to root caries, especially if plaque control was suboptimal. Antibiotics appeared to weaken the association between plaque levels and the abundance of *Selenomonas* and *Leptotrichia* (Table 2). Individuals in the adjunctive antibiotic group however exhibit also elevated *Selenomonas* and *Leptotrichia* levels compared to the placebo group (Suppl. Figure S3), suggesting a shift toward a more cariogenic subgingival reservoir. This may imply that antibiotic exposure may select for acidogenic taxa independently of plaque accumulation, potentially increasing caries risk.

*Selenomonas* and *Leptotrichia* species play a complex role in dental plaque and periodontal health. While some *Selenomonas* species are linked to severe early childhood caries [39], their overall prevalence in periodontal disease is comparatively low [43]. *Selenomonas* spp. contribute significantly to biofilm structure in periodontal pockets [43] and are associated to caries in children with low *Streptococcus mutans* levels [39]. *Selenomonas sputigena* can become enmeshed within *S. mutans*-derived exopolysaccharides, forming a honeycomb-like microcolony architecture that amplifies acid production and lesion severity [44]. Furthermore, certain *Selenomonas* species correlate positively with probing depth in aggressive periodontitis [45]. During periods of oral hygiene discontinuation, there is a notable increase in the relative abundance of *Leptotrichia* species in both supragingival plaque and saliva samples, which may contribute to tooth decay due to their ability to ferment carbohydrates and produce lactic acid [46,47]. While *Leptotrichia* are part of the normal oral microbiota, they can act as opportunistic pathogens in various diseases, particularly in immunocompromised individuals, necessitating accurate species identification for proper diagnosis and treatment [47].

Consistently, both *Selenomonas* and *Leptotrichia* have been identified as caries-associated pathobionts in adults, particularly in root surface lesions [48]. Interestingly, in young individuals with caries lesions higher abundances of *Streptococcus mutans* are found compared to *Selenomonas-,* and *Leptotricha* ssp. [49,50]. Their abundance in plaque or saliva could potentially signal early shifts toward cariogenic conditions, enabling timely preventive interventions [37]. Novel approaches, including enzyme-based disruption of biofilm architecture or probiotic supplementation designed to alter the plaque microenvironment, might specifically undermine their pathogenic synergy, providing additional tools for managing caries risk in adult and elderly populations. Future studies should further explore and validate these microbial targets to refine individualized prevention and treatment strategies.

As this study was originally designed to assess the efficacy of adjunctive antibiotics on clinical parameters of periodontitis there are some limitations as the study protocol was not optimized for this specific research question. For instance, the variability in pre-treatment protocols across study centers, which have influenced baseline plaque levels. Furthermore, as the initial study was focused on periodontal health no caries assessment was done, so we can only speculate if the findings may contribute to caries risk assessment. Future studies focusing on this research question should aim to standardize pre-treatment protocols to minimize variability and enhance the comparability of results. Our short read approach using the V4 hypervariable region of the 16S-RNA gene limits to genus level resolution, Therefore, the use of advanced microbial sequencing techniques with longer read lengths and robust species level resolution could provide deeper insights into the specific microbial species associated with plaque formation and their potential roles in periodontal health.

## Conclusion

In conclusion, our findings demonstrate that supragingival plaque levels are significantly associated with subgingival microbial characteristics in stage III – IV periodontitis patients. High plaque values were linked to increased bleeding on probing, greater microbial richness, and the emergence of a distinct microbial topic characterized by *Selenomonas* and *Leptotrichia*. Importantly, adjunctive antibiotic therapy moderated this association, underscoring its potential role in personalized periodontal treatment strategies. This modulation may reflect a shift toward a more cariogenic subgingival profile, even in patients with lower plaque levels, highlighting the need for closer examination. Future studies employing species-level resolution are warranted to further elucidate the intricate interactions between supragingival plaque management and subgingival microbiota during periodontal therapy.

## Supplementary Material

Suppl_Figures_Saberi.pdf

Suppl Table 1.xlsx
