# Supplementary material for: Association of supragingival plaque management with subgingival microbiota is moderated by adjunctive antibiotics in stage III-IV periodontitis patients during periodontal therapy
Source: J Oral Microbiol. 2025 Jun 14;17(1):2517043. doi: 10.1080/20002297.2025.2517043 (PMC12168411; doi:10.1080/20002297.2025.2517043)
Supplement: Suppl_Figures_Saberi.pdf [file ZJOM_A_2517043_SM6997.pdf]

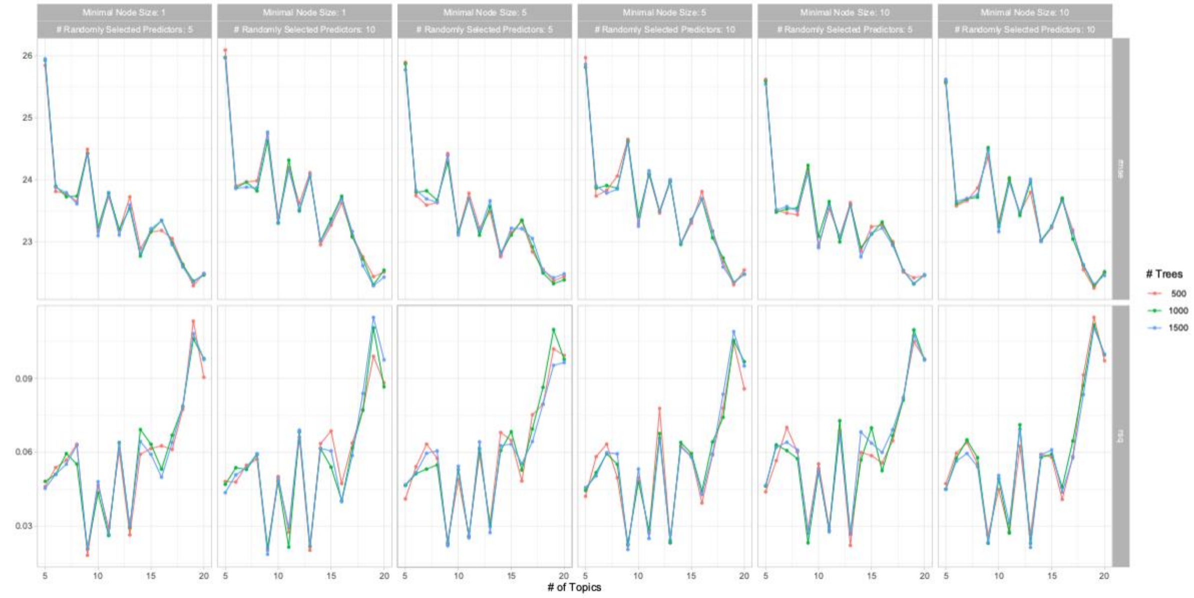

Suppl. Fig. 1.: Performance metrics of cross-validated random forest models across different parameter spaces, based on LDA transformed microbiome data. First row shows root mean-squared error (RMSE), second row shows R^2.

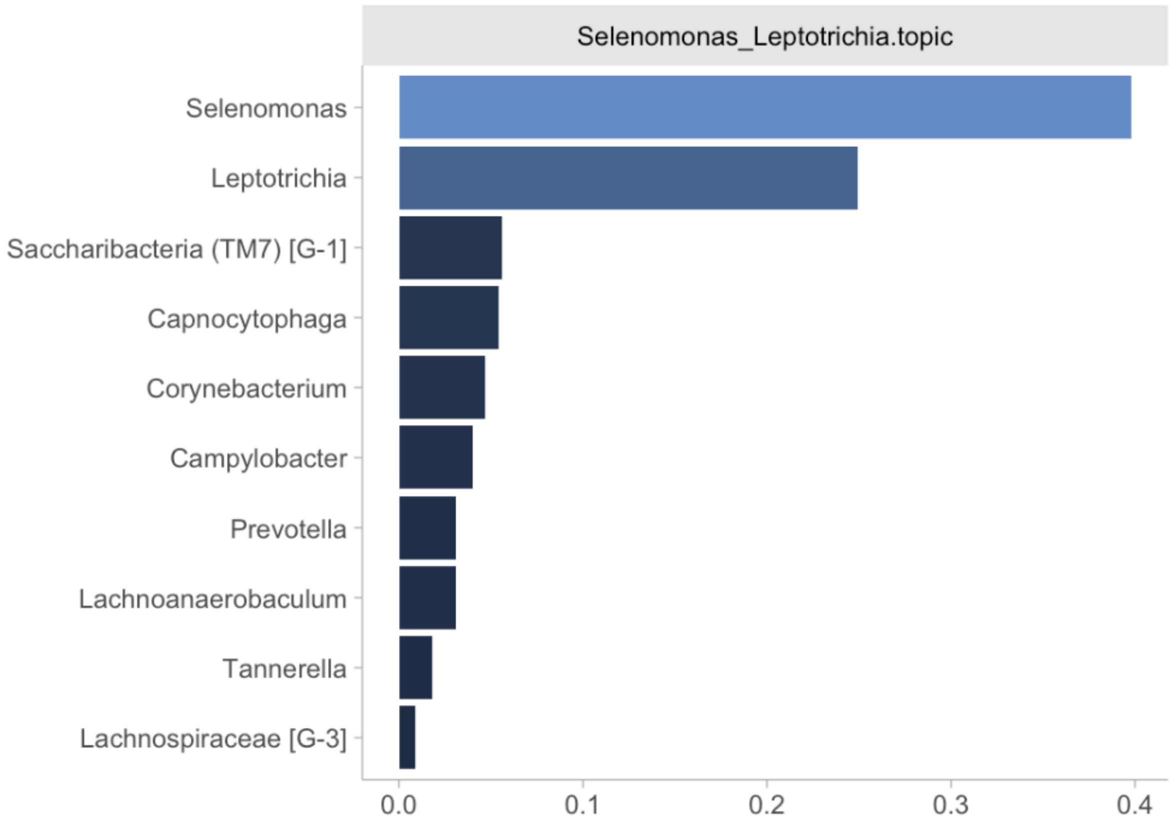

Suppl. Fig. 2: Relative Abundance of Bacterial Genera in the Selenomonas Leptotrichia Topic. This bar chart displays the relative abundances of various bacterial genera associated with the Selenomonas Leptotrichia topic. The x-axis represents the proportion of each genus, while the y-axis lists the genera in descending order of abundance.

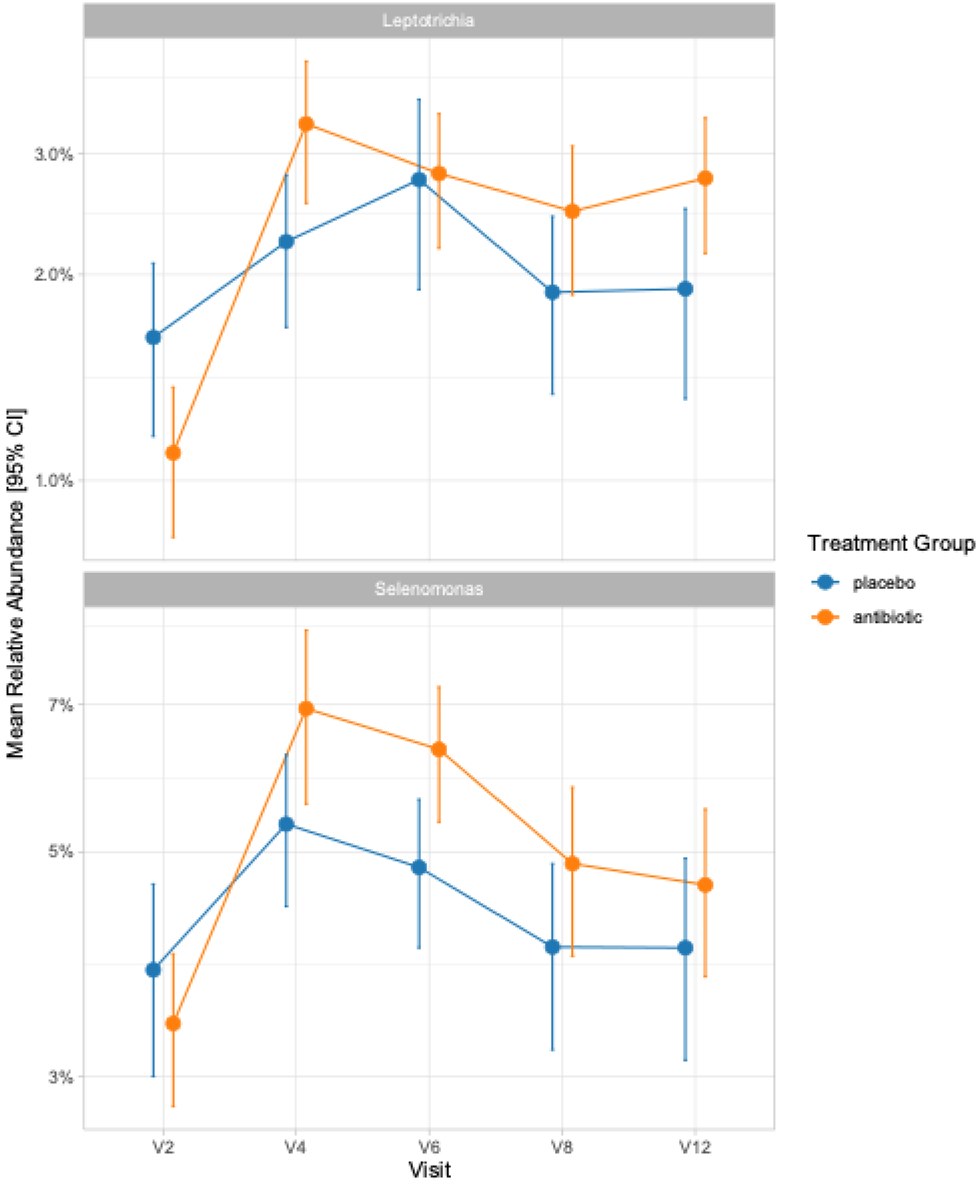

Supp. Fig. 3: Temporal dynamics in mean abundance of genera *Leptotrichia* and *Selenomonas*. Error bars represent 95% Confidence Intervals (CI).

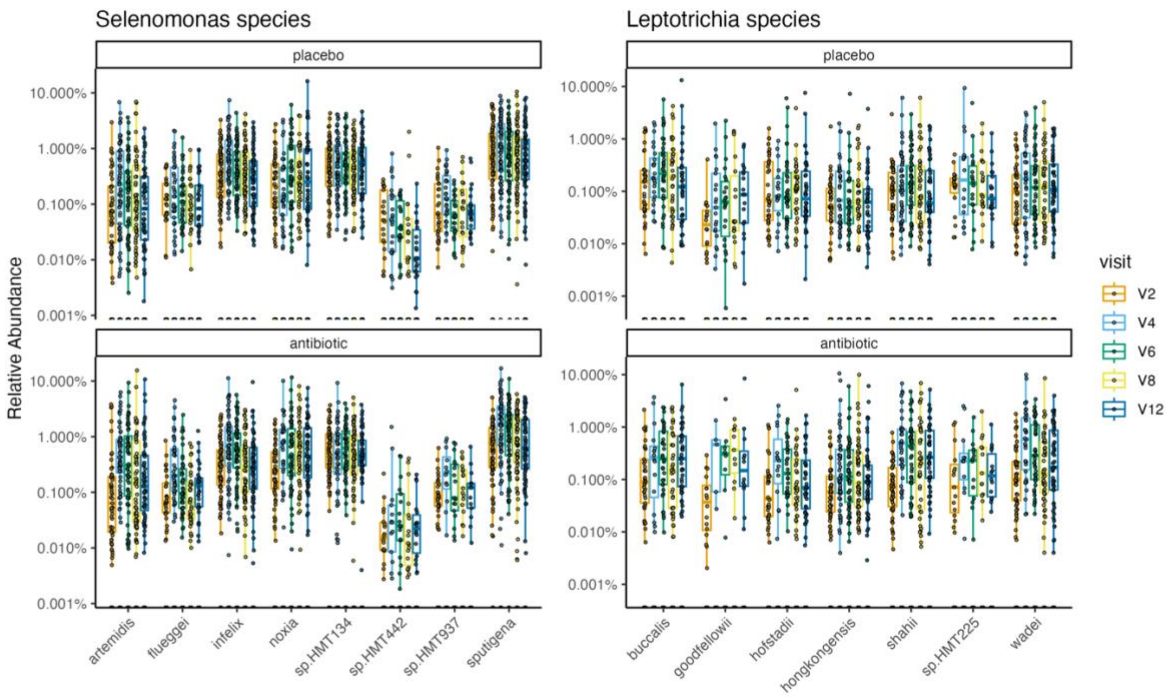

Suppl. Fig 4: Log-Relative abundance of *Selenomonas* and *Leptotrichia* species across different visits in placebo and antibiotic treatment groups. Data are stratified by treatment (placebo in upper panels, antibiotic in lower panels) and colored by visit timepoint (V2 - before treatment, V4 - two months after treatment, V6 eight months after treatment, V8 – 14 months after treatment, and V12 – 26 months after treatment).
